# Understanding biochemistry: structure and function of nucleic acids

**DOI:** 10.1042/EBC20180038

**Published:** 2019-10-11

**Authors:** Steve Minchin, Julia Lodge

**Affiliations:** School of Biosciences, University of Birmingham, Birmingham, United Kingdom

**Keywords:** Concept of the gene, DNA Replication, Nucleic acids, Transcription, Translation

## Abstract

Nucleic acids, deoxyribonucleic acid (DNA) and ribonucleic acid (RNA), carry genetic information which is read in cells to make the RNA and proteins by which living things function. The well-known structure of the DNA double helix allows this information to be copied and passed on to the next generation. In this article we summarise the structure and function of nucleic acids. The article includes a historical perspective and summarises some of the early work which led to our understanding of this important molecule and how it functions; many of these pioneering scientists were awarded Nobel Prizes for their work. We explain the structure of the DNA molecule, how it is packaged into chromosomes and how it is replicated prior to cell division. We look at how the concept of the gene has developed since the term was first coined and how DNA is copied into RNA (transcription) and translated into protein (translation).

## The structure of deoxyribonucleic acid

Deoxyribonucleic acid (DNA) is one of the most important molecules in living cells. It encodes the instruction manual for life. Genome is the complete set of DNA molecules within the organism, so in humans this would be the DNA present in the 23 pairs of chromosomes in the nucleus plus the relatively small mitochondrial genome. Humans have a diploid genome, inheriting one set of chromosomes from each parent. A complete and functioning diploid genome is required for normal development and to maintain life.

### Discovery and chemical characterisation of DNA

DNA was discovered in 1869 by a Swiss biochemist, Friedrich Miescher. He wanted to determine the chemical composition of leucocytes (white blood cells), his source of leucocytes was pus from fresh surgical bandages. Although initially interested in all the components of the cell, Miescher quickly focussed on the nucleus because he observed that when treated with acid, a precipitate was formed which he called ‘nuclein’. Almost all molecular bioscience graduates would have repeated a form of this experiment in laboratory classes where DNA is isolated from cells. Miescher, Richard Altmann and Albrecht Kossel further characterised ‘nuclein’ and the name was changed to nucleic acid by Altmann. Kossel went on to show that nucleic acid contained purine and pyrimidine bases, a sugar and phosphate. Work in the 1930s from many scientists further characterised nucleic acids including the identification of the four bases and the presence of deoxyribose, hence the name deoxyribonucleic acid (DNA). Erwin Chargaff had found that DNA molecules from a particular species always contained the same amount of the bases cytosine (C) and guanine (G) and the same amount of adenosine (A) and thymine (T). So, for example, the human genome contains 20% C, 20% G, 30% A and 30% T.

**Figure 1:**
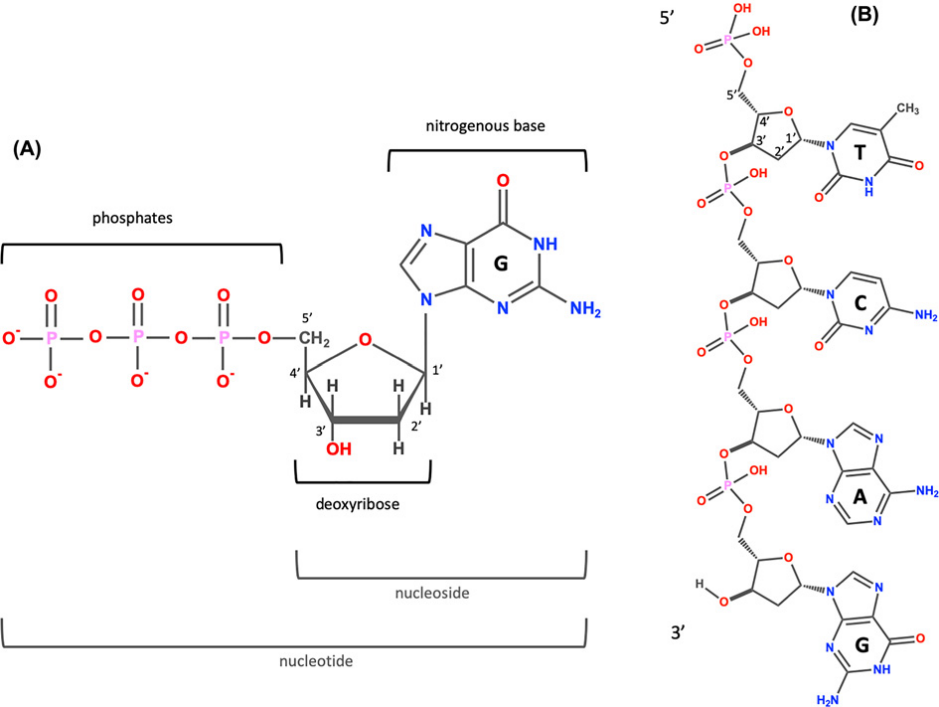
The structure of DNA (**A**) A nucleotide (guanosine triphosphate). The nitrogenous base (guanine in this example) is linked to the 1′ carbon of the deoxyribose and the phosphate groups are linked to the 5′ carbon. A nucleoside is a base linked to a sugar. A nucleotide is a nucleoside with one or more phosphate groups. (**B**) A DNA strand containing four nucleotides with the nitrogenous bases thymine (T), cytosine (C), adenine (A) and guanine (G) respectively. The 3′ carbon of one nucleotide is linked to the 5′ carbon of the next via a phosphodiester bond. The 5′ end is at the top and the 3′ end at the bottom.

DNA is a polymer made of monomeric units called nucleotides (Figure 1A), a nucleotide comprises a 5-carbon sugar, deoxyribose, a nitrogenous base and one or more phosphate groups. The building blocks for DNA synthesis contain three phosphate groups, two are lost during this process, so the DNA strand contains one phosphate group per nucleotide.

There are four different bases in DNA, the double-ring purine bases: adenine and guanine; and the single-ring pyrimidine bases: cytosine and thymine (Figure 1B). The carbon within the deoxyribose ring are numbered 1′ to 5′. Within each monomer the phosphate is linked to the 5′ carbon of deoxyribose and the nitrogenous base is linked to the 1′ carbon, this is called an N-glyosidic bond. The phosphate group is acidic, hence the name nucleic acid.

In the DNA chain (Figure 1B), the phosphate residue forms a link between the 3′-hydroxyl of one deoxyribose and the 5′-hydroxyl of the next. This linkage is called a phosphodiester bond. DNA strands have a ‘sense of direction’. The deoxyribose at the top of the diagram in Figure 1B is not linked to another deoxyribose; it terminates with a 5′ phosphate group. At the other end the chain terminates with a 3′ hydroxyl.

### DNA is the genetic material

Although many scientists, including Miescher, had observed that prior to cell division the amount of nucleic acid increased, it was not believed to be the genetic material until the work of Fredrick Griffith, Oswald Avery, Colin MacLeod and Maclyn McCarty. In 1928, Griffith showed that living cells could be transformed by extracts from heat-killed cells and that this transformation had the potential to permanently change the genetic makeup of the recipient cell. Griffith was working with two strains of the bacterium *Streptococcus pneumoniae.* The encapsulated so-called S strain is virulent, whereas the non-capsulated R strain is nonvirulent. If the S strain is injected subcutaneously into mice, the mice die, whereas, if either live R strain is injected or heat-killed S strain is injected, the mouse lives. However, if a mixture of live R strain and heat-killed S strain is injected into a mouse, the mouse will die, and live S strain can be isolated from the blood. So, in the Griffith experiment a component of the heat-killed S strain is transforming the R strain. In 1944, Avery, MacLeod and McCarty went on to show that it was DNA that could transform the avirulent bacterium. They isolated a crude DNA extract from the S strain and destroyed any protein, lipid, carbohydrate and ribonucleic acid (RNA) component and showed that this purified DNA could still transform the R strain. However, when the purified DNA was treated with DNAse, an enzyme that degrades DNA, transformation was lost.

Alfred Hershey and Martha Chase confirmed that DNA was the genetic material. They used a virus that infects bacteria called a bacteriophage. The bacteriophage contains a protein capsid surrounding a DNA molecule. They showed that when bacteriophage T2 infects *Escherichia coli*, it is the phage DNA, not protein, that enters the bacterial cell.

### Determining the structure of DNA

**Figure 2:**
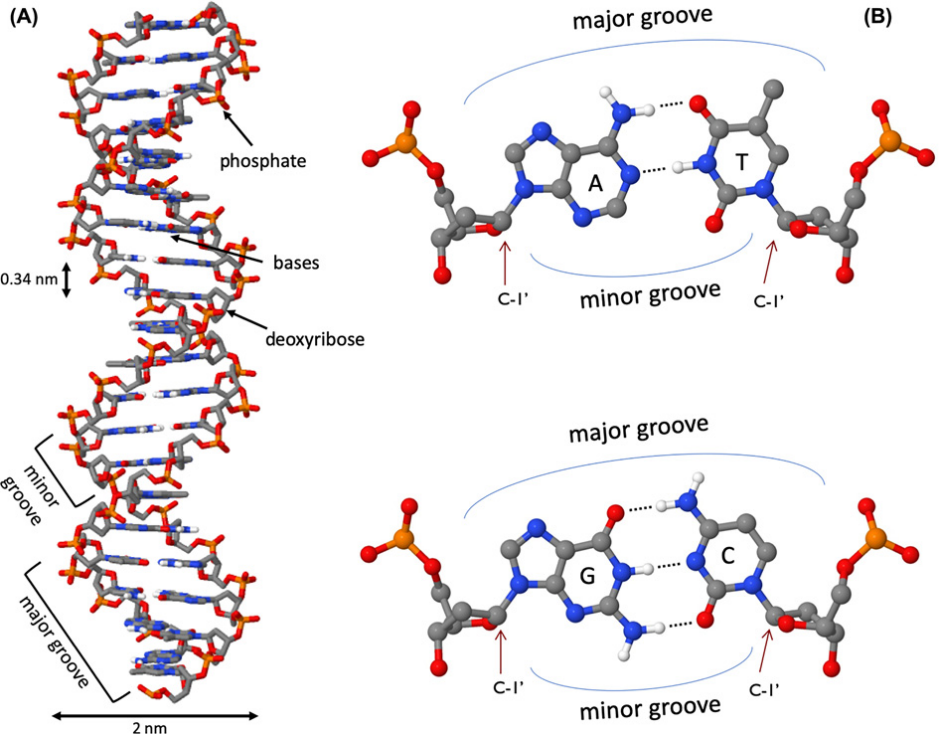
DNA structure (**A**) The DNA double helix, with the sugar phosphate backbone on the outside and the nitrogenous bases in the middle. (**B**) An A:T and a G:C base pair with the C1′ of the deoxyribose indicated by the arrow. Note that the C1′ of the deoxyribose is in the same position in all base pairs. In this figure, the atoms on the upper edge of the base pair face into the major groove and those facing lower edge face into the minor groove. The hydrogen bonds between the base pairs are indicated by the dotted line.

Once it had been shown that DNA was the genetic material, there was a race to determine the three-dimensional structure of the DNA molecule. At King’s College London, Rosalind Franklin and Maurice Wilkins, having obtained data using X-ray diffraction, had proposed that DNA had a helical structure and Franklin had obtained a particularly good X-ray diffraction pattern. In Cambridge, James Watson and Francis Crick used model building together with data from a variety of sources including Franklin’s X-ray diffraction pattern and Chargaff’s base composition data to work out the now well-known double helix structure of DNA. Their work was published in *Nature* in 1953. The Watson–Crick structure is shown in Figure 2A.

DNA is a two-stranded helical structure, the two strands run in opposite directions. In Figure 2A, one strand is running 5′ to 3′ top to bottom, whereas the other strand is running 3′ to 5′ top to bottom. The helix is right-handed which means that if you are looking down the axis, the helix turns clockwise as it gets further away from you. The two chains interact via hydrogen bonds between pairs of bases with adenine always pairing with thymine, and guanine always pairing with cytosine. The Watson–Crick structure therefore accounts for and explains the Chargaff data which showed that there was always an equal amount of C and G and of A and T. The regular nature of the double helix comes about because the distance between the 1′ carbon of the deoxyribose on one strand and 1′ carbon of the opposite deoxyribose is always the same irrespective of the base pair (Figure 2B). The 1′ carbons of the deoxyribose opposing nucleotides do not lie directly opposite each other on the helical axis, this means that the two sugar–phosphate backbones are not equally spaced along the helical axis resulting in major and minor grooves.

The diameter of the helix is 2 nm, adjacent bases are separated by 0.34 nm (0.34 × 10^−9^ m) and related by a rotation of 36°, this results in the helical structure repeating every 10 residues. DNA molecules are normally very long and the sequence of bases along the DNA chain is not restricted. For example, the genome of the bacterium *E. coli* is a single circular chromosome which contains 4.6 million base pairs (4.6 × 10^6^ bp), this is therefore 1.6 mm long (4.6 × 10^6^ × 0.34 × 10^−9^ m). The human genome is made up of 24 distinct chromosomes, chromosomes 1–22 and the X and Y chromosomes present in the nucleus plus mitochondrial DNA. The nuclear chromosomes vary in size from approximately 50–250 × 10^6^ bp, the mitochondrial DNA is 17 × 10^3^ bp. The total length of a haploid human genome is 3 × 10^9^ bp. Within a single human diploid cell, which contains 23 chromosome pairs there is 2 m of DNA. Based on the assumption that humans contain 3 trillion cells with a nucleus, if all the DNA from a single human individual was put end to end, it would reach to the sun and back approximately 20 times.

### RNA

**Figure 3:**
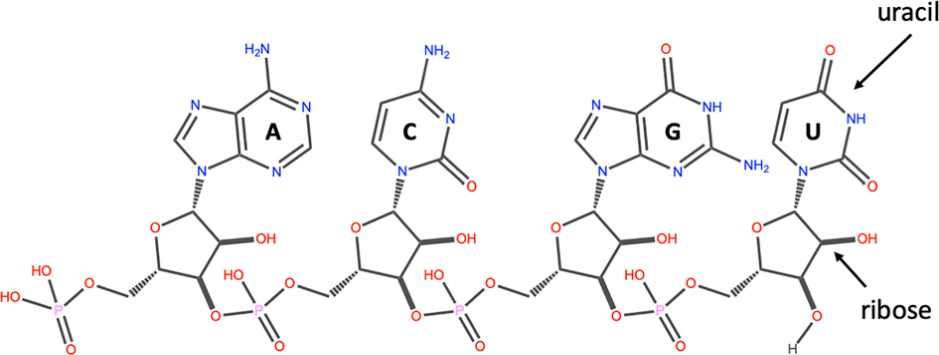
The structure of RNA An RNA strand containing the four nucleotides with the nitrogenous bases: adenine (A), cytosine (C), guanine (G) and uracil (U) respectively. The 3′ carbon of the ribose of one nucleotide is linked to the 5′ carbon of the next via a phosphodiester bond. The 5′ end on the left and the 3′ end on the right.

Another important class of nucleic acids is RNA, the roles of RNA molecules in the cell will be discussed below. Chemically RNA is similar to DNA, it is a chain of similar monomers. The building blocks are nucleotides containing the 5-carbon sugar ribose, a phosphate and a nitrogenous base. The phosphate is attached to the 5′ carbon of the ribose and the nitrogenous base to the 1′ carbon (Figure 3). RNA contains four bases adenine, guanine, cytosine and uracil. RNA is more labile (easily broken down) than DNA and most RNA molecules do not form stable secondary structures, some notable exceptions will be discussed below. The properties of RNA make it ideal as a genetic messenger during protein synthesis, the idea of this genetic messenger, mRNA, was proposed by François Jacob and Jacques Monod.

### Packaging of DNA into eukaryotic cells

**Figure 4:**
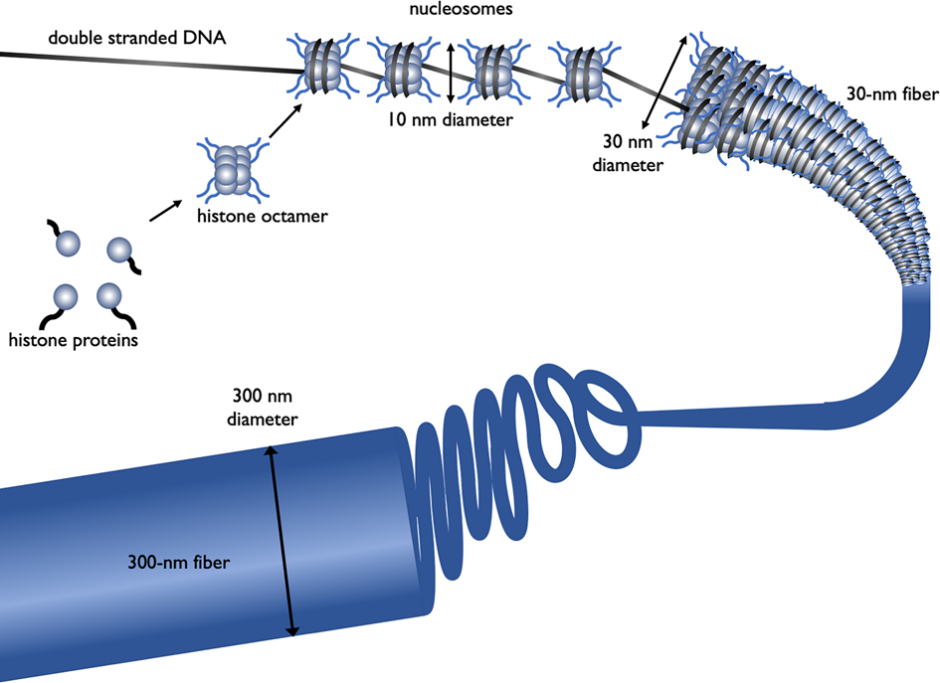
The different levels of chromatin structure Histone proteins (H2A, H2B, H3 and H4) associate to form a histone octamer. Approximately 147 bp of DNA wraps around histone octamer to form a nucleosome, generating a ‘beads on a string’ structure, the nucleosome together with histone H1 condense into the 30-nm fibre, there is further condensation to form the 300-nm fibre. During mitosis there is further compaction (not shown).

DNA has to be highly condensed to fit into the bacterial cell or eukaryotic nucleus. In eukaryotes, histone proteins are used to condense the DNA into chromatin. The basic structure of chromatin is the nucleosome, a nucleosome contains DNA wrapped almost two times around the histone octamer (comprising two copies each of the histone proteins H2A, H2B, H3 and H4) (Figure 4). Further levels of compaction are required to fit the DNA into the nucleus (Figure 4), the nucleosomes are folded upon themselves to form the 30-nm fibre, this is then folded again to form the 300-nm fibre and during mitosis further compaction can occur forming the chromatid which is 700 nm in diameter.

Processes such as DNA replication and DNA transcription need to occur in the chromatin environment and because of the level of compaction, this acts as a barrier to proteins that need to interact with DNA. Therefore, chromatin structure plays an important role in processes such as regulation of gene expression in eukaryotes. DNA and the histone proteins can be chemically modified, these are called epigenetic modifications as they do not change the DNA sequence, however, they can be passed on during cell division and to subsequent generations, a process known as epigenetic inheritance. As these epigenetic modifications can alter the chromatin structure they regulate gene transcription and can affect the phenotype. Epigenetics plays key roles in many processes, including development, cancer and behaviour and addiction. This will be discussed further later in this article.

Nuclear organisation plays an important role in many biological processes including regulation of gene transcription. In recent years the development of several techniques, including microscopy, have allowed us to gain an understanding of the way the genome is organised in 3D. Individual chromosomes are not randomly spaced within the nucleus; each chromosome has a distinct territory. Actively transcribed regions from different chromosomes are often close to each other and near the interior of the nucleus, whereas, inactive genes are on the periphery or near a special area called the nucleolus where ribosomal RNA is transcribed.

## DNA replication

Whenever a cell divides there is a need to synthesise two copies of each chromosome present within the cell. For example in a human, prior to cell division, all 23 pairs of chromosomes need to be replicated to form 46 pairs, so that following cell division each daughter cell has a full complement (23 pairs) of chromosomes. The structure of DNA gives us a clue to how it is replicated, this was eloquently postulated by Watson and Crick in their 1953 paper: “It has not escaped our notice that the specific pairing we have postulated immediately suggests a possible copying mechanism for the genetic material”. Each strand can act as a template for the synthesis of the complementary strand, so the replication machinery would ‘unzip’ the double helix and read along the two existing ‘parent’ strands, synthesising a complementary new ‘daughter’ strand with A opposite T, C opposite G etc. This is described as semi-conservative, since each ‘new’ double-stranded DNA molecule has one original parent strand and one newly made daughter ‘strand’.

The evidence that DNA replication was semi-conservative came from an elegant experiment completed by Matthew Meselson and Franklin Stahl. They labelled the parental DNA with a heavy isotope of nitrogen (^15^N) by growing bacteria in a growth medium that contained ^15^NH_4_Cl. They then grew the bacteria, in a medium that contained ^14^NH_4_Cl, in conditions such that any newly synthesised DNA would contain ^14^N. Since DNA replication is semi-conservative, after one round of DNA replication, each cell would have a DNA molecule that contains one ‘old’ parental strand labelled with ^15^N and one ‘new’ daughter strand labelled with ^14^N. This was shown by analysing the density of the DNA using density-gradient centrifugation. As predicted, they observed that the new daughter DNA molecule had a density consistent with the fact that it contained both ^15^N and ^14^N and that this daughter DNA contained one strand with ^15^N and another strand with ^14^N.

### DNA polymerase and DNA synthesis

**Figure 5:**
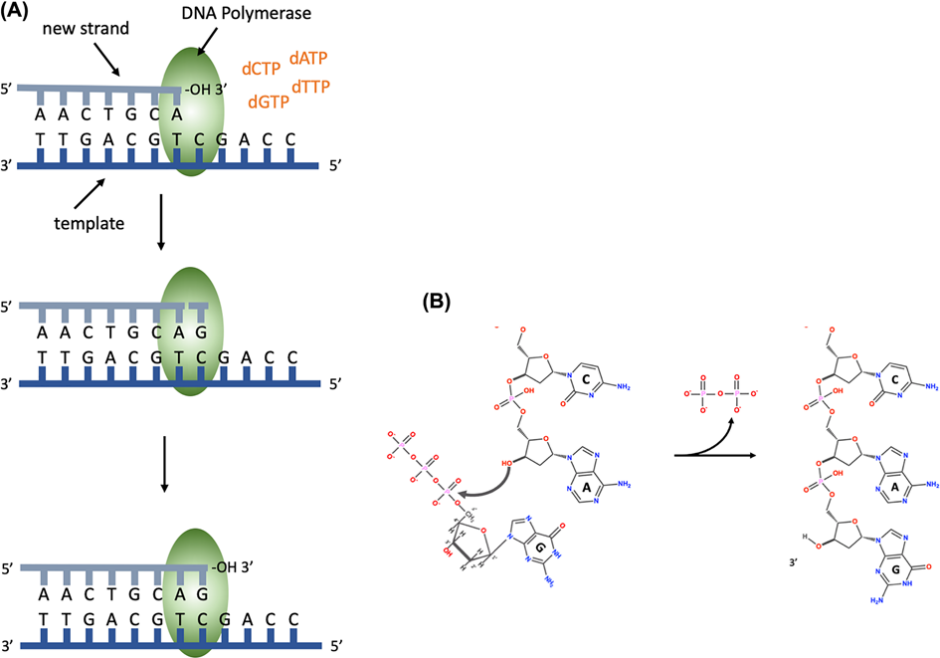
DNA synthesis (**A**) DNA polymerase binds the template DNA and the new strand. The next nucleotide to be added to the 3′ end of the growing chain will contain guanine (G), this is complementary to the C on the template strand. DNA polymerase catalyses the formation of a phosphodiester bond. (**B**) The chemical reaction during the formation of a phosphodiester bond, showing the addition of a nucleotide containing guanine and the release of pyrophosphate.

The enzyme, DNA polymerase, is responsible for DNA synthesis. DNA polymerase is a template-driven enzyme, so it will use the parental DNA strand as a template. It cannot synthesise DNA in the absence of a template. In addition, it will only add nucleotides on to the 3′ end of an existing nucleic acid chain. The building blocks for DNA synthesis are deoxynucleoside triphosphates (dATP, dTTP, dCTP and dGTP). During DNA synthesis, the base within the incoming deoxynucleoside triphosphate pairs with the complementary base on the template strand, a phosphodiester bond is formed between the 5′ phosphate on the incoming nucleotide and the free 3′ hydroxyl on the existing nucleic acid chain; pyrophosphate is released (Figure 5).

Pyrophosphate is the two phosphate residues within the deoxynucleoside triphosphate building block that are not incorporated into the DNA chain. DNA polymerase synthesises DNA in the 5′ to 3′ direction, because it can only add nucleotides on to the 3′ end of the chain. DNA polymerase has proofreading activity, so after the phosphodiester bond has been formed, the base pairing is checked and if a nucleotide with an incorrect base has been added, DNA polymerase will remove the nucleotide using a 3′ to 5′ exonuclease activity. Exonucleases are enzymes that can remove nucleotides from the ends of a DNA molecule, 3′ to 5′ exonucleases remove nucleotides from the 3′ end of a DNA molecule and therefore can remove the last nucleotide that was added during DNA replication. This is analogous to using the delete key to remove a letter that you have typed incorrectly before adding the correct one and continuing typing.

DNA polymerase requires a short double-stranded region with a free 3′ hydroxyl in order to start making a copy of the template; this ensures that DNA is synthesised in a controlled way. Initiation of DNA synthesis uses a small RNA primer (8–12 bases) made by the enzyme primase. DNA polymerase will then extend from the primer copying the template and synthesising the daughter DNA strand. This means that when DNA synthesis first starts each DNA molecule actually contains a small piece of RNA at its 5′ end. This RNA will ultimately be replaced with DNA, how this is done is discussed below.

### The origin of replication and the replisome

**Figure 6:**
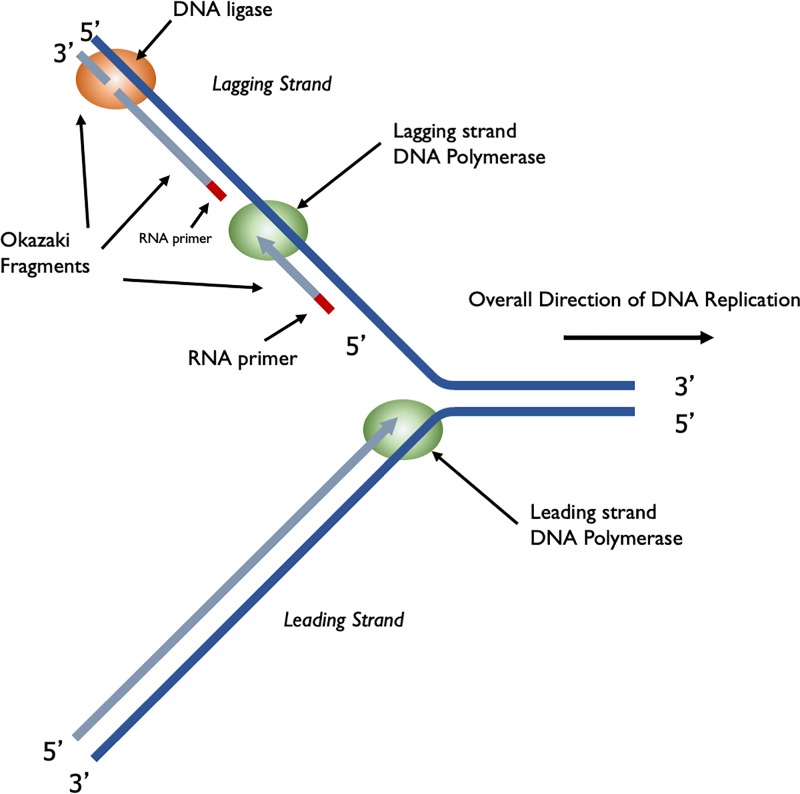
DNA synthesis at a replication fork A single replication fork showing the leading and lagging strands. The leading strand is synthesised continuously, reading the template 3′ to 5′, synthesising DNA in the 5′ to 3′ direction. The lagging strand is synthesised discontinuously, in short Okazaki fragments (1000 bases in prokaryotes and 100 bases in eukaryotes).

A large multiprotein complex, called the replisome, is responsible for DNA replication. In prokaryotes, two replisomes form at a specific point on the chromosome called the Origin of Replication (*ori*). The DNA in this region will be opened up, ‘unzipped’ so that the replication machinery can gain access to single-stranded parental DNA, which will act as template for synthesis of the new daughter strands. The two replisomes then travel in opposite directions around the circular prokaryotic chromosome, each replisome forming a replication fork, a schematic representation of one replication fork is shown in Figure 6.

### The replication fork

Within the replication fork, on the so-called leading strand, DNA polymerase moves 3′ to 5′ with respect to the template and synthesises DNA in the 5′ to 3′ direction as it moves in the same direction as the replication fork. Although overall the lagging strand is synthesised in the 3′ to 5′ direction, it is actually synthesised discontinuously in small segments called Okazaki fragments, which are synthesised 5′ to 3′ (Figure 6). Each Okazaki fragment will be started with an RNA primer and is synthesised in the opposite direction to the movement of the replication fork. In prokaryotes, Okazaki fragments are 1000–2000 bases in length. In Figure 6 you will see that the DNA polymerase synthesising the Okazaki fragment will eventually reach the primer for the previous Okazaki fragment. When this happens the primer for the previous fragment is removed by a DNA polymerase using 5′ to 3′ exonuclease activity. DNA polymerase then replaces the missing nucleotides by adding them to the 3′ end of the last Okazaki fragment. When all the primer has been removed, there will be two DNA strands adjacent to each other but not joined by a phosphodiester bond, these two strands are joined together by the enzyme DNA ligase.

The replisome contains a number of other important proteins required for DNA replication. The double-stranded DNA needs to be separated, ‘unzipped’, by a helicase to generate the single-stranded DNA templates for DNA polymerase. As the replication fork moves along the helical DNA, the coils in the DNA in front of the fork become compressed so the DNA is described as being overwound; a topoisomerase is required to ‘relax’ it by remove the over-winding. Single-stranded binding proteins (SSBs) bind the lagging strand template to stabilise and protect the single-stranded DNA.

The two replication forks that form at the *ori* will move in opposite directions around the circular prokaryotic genome until they reach the terminator sequence, *ter*, which is on the opposite side of the genome compared with the *ori*, i.e. it is at 6 o’clock compared with 12 o’clock. This results in the complete replication of the genome. Once DNA replication has been completed a post-replication DNA repair process will correct errors that were not corrected by the proofreading activity of DNA polymerase. The fidelity of DNA replication is extremely high, resulting in an error rate of 1 mistake per 10^9^–10^10^ nucleotides added.

### DNA replication in eukaryotes

DNA replication is essentially the same in eukaryotes and prokaryotes. In both cases two replisomes form at an *ori* and generate two replication forks moving in opposite directions away from the origin. In each replication fork there are leading and lagging strands. There are two major differences. The first is that, due to the larger genome size, each chromosome has multiple origins of replication, so there will be a large number of replication forks on each chromosome.

**Figure 7:**
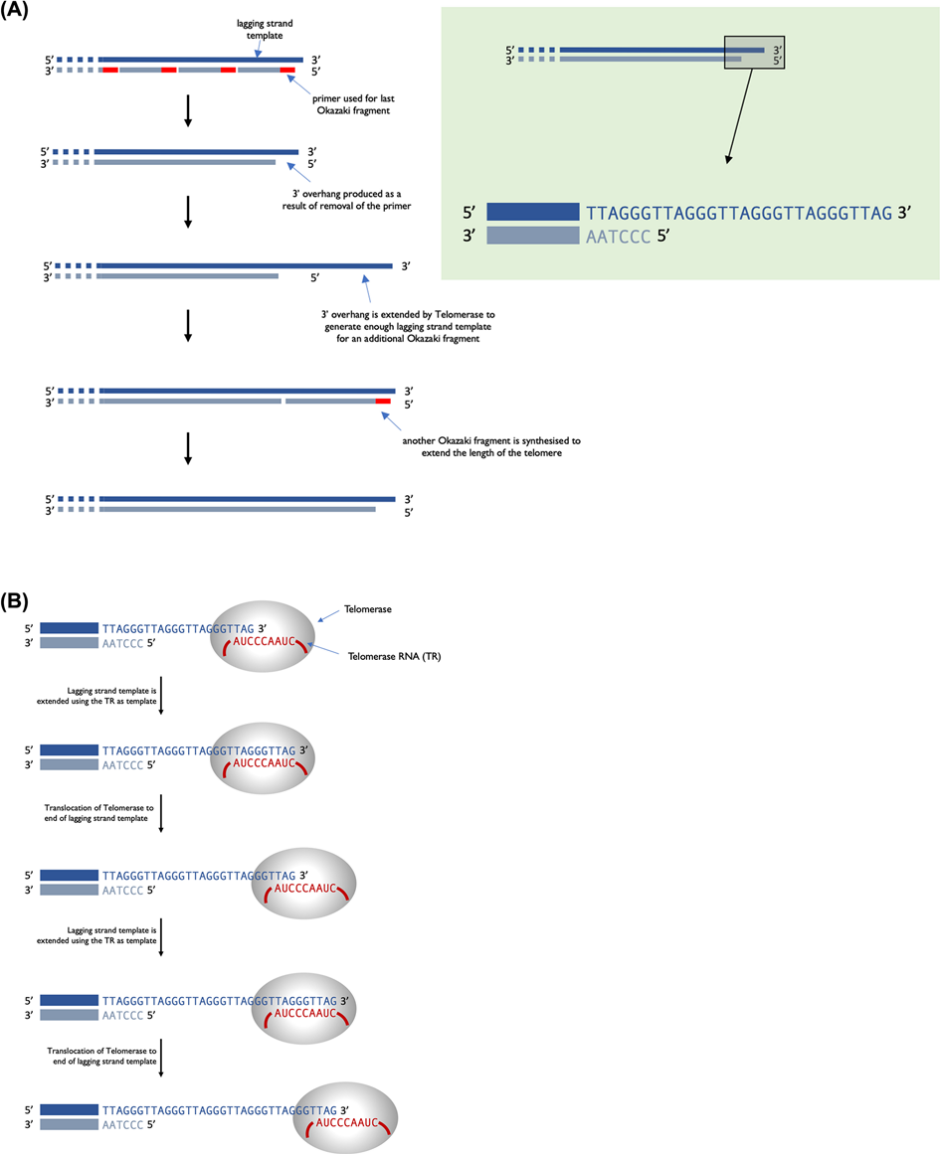
Telomeres and telomerase (**A**) Following DNA replication and removal of the primer for the last Okazaki fragment of the lagging strand, there will be a region at the 3′ end that is not base paired, called a 3′ overhang. (**B**) Telomerase binds and uses the RNA it contains to act as a template to extend the 3′ overhang. This extends the 3′ end sufficiently for a new RNA primer to bind and the final Okazaki fragment to be made.

The second difference is that, with the exception of mitochondrial DNA, eukaryotic chromosomes are linear and this results in an issue because of lagging strand synthesis. Replication of a linear chromosome results in shortening of one 5′ end of each daughter DNA molecule. This is because when the primer required for the last Okazaki fragment is removed, DNA polymerase cannot fill the gap (Figure 7A). Repeated rounds of DNA replication results in shorter and shorter DNA molecules. If this is not corrected, eukaryotes would have become extinct as their chromosomes get shorter with each generation. Eukaryotes have a mechanism to preserve the ends of chromosomes when it counts; that is in the gametes. The terminal ends of chromosomes, telomeres, contain a highly repeated sequence, for example, in humans the sequence TTAGGG is repeated in tandem 100 to over 1000-times. Repeated rounds of DNA replication will result in the shortening of these telomeric sequences that is the number of repeats will reduce. Telomerase, an RNA containing enzyme, can add additional copies of the repeat sequence to the 3′ end, replacing those lost during DNA replication (see Figure 7).

This actually extends the 3′ end of the telomere rather than extending the 5′ that is initially lost during DNA replication. The RNA sequence within telomerase is complementary to the 3′ telomeric sequence and so can bind and act as a template for synthesis of a short DNA sequence. Telomerase then moves along the newly synthesised strand and the process is repeated. Multiple rounds of elongation and translocation ultimately results in the 3′ end being extended so that it is long enough for it to act as template for synthesis of another Okazaki fragment, hence extending both strands of the telomere. Only germ cells and a few other actively dividing cells (e.g. haematopoietic cells) have sufficient levels of telomerase activity to counteract the loss of repeat sequences during DNA replication. At birth, telomeres are over 10000 base pairs in length and there are enough repeats to allow DNA replication and somatic cell division during the lifetime of the organism. If telomeres become too short this will trigger programmed cell death (a process called apoptosis). The lack of telomerase activity in somatic cells limits the number of cell divisions that can occur, and this is a ‘problem’ that needs to be overcome by cancer cells. Telomerase activity is reactivated in most cancers, allowing these cells to divide indefinitely and therefore this activity is a potential target for cancer therapies.

An understanding of DNA synthesis is central to many experimental approaches in molecular biosciences, it allows us to determine DNA sequences including that of the human genome, to analyse environmental samples to better understand the living world around us and to analyse minute biological samples from crime scenes to identify offenders. It is exploited in medicine, for example several drugs used to treat HIV infection or exposure are nucleoside analogues that inhibit DNA synthesis. Many chemotherapy agents used to treat cancer target DNA replication.

## The genetic code and the concept of a gene

As we have seen in the previous two sections, the genetic material in a cell is made of DNA and can be copied and passed on to progeny through DNA replication allowing for inheritance of the information that it carries. A large proportion of the information on the DNA is first transcribed into mRNA and then translated into proteins. However there are some RNAs that are never translated into proteins and these have important functions too. Phrases like ‘it is in my genes’ or ‘in my DNA’ are used in common speech to mean to be an important part of who someone is.

**Figure 8:**
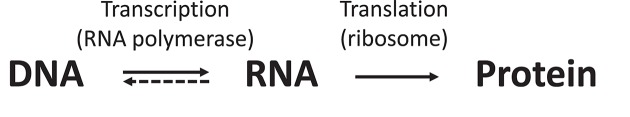
The flow of genetic information The arrows represent steps where DNA or RNA is being used as a template to direct the synthesis of another polymer, either RNA or protein.

The term gene was coined in the early 1900s to describe the basic unit of heredity. Genes were thought of as distinct loci arranged lineally on chromosomes. Breeding experiments with the fruitfly *Drosophila* supported this view and showed that if two genes are close together on a chromosome they are more likely to be inherited together. The observation that mutations in genes could give rise to altered phenotypes gave rise to the ‘one gene one polypeptide’ hypothesis. Once it became clear that genes were made of DNA, what is referred to as the central dogma of molecular biology was coined. This describes a two step process in which the genes on the DNA are transcribed into RNA and then translated into a sequence of amino acids that makes up a protein. The information flow is from DNA to RNA and then to protein (Figure 8).

However there are exceptions to this, firstly some viruses have RNA genomes and in some cases these are reverse transcribed into DNA before the genes can be expressed. The retrovirus HIV is an example of this. The other exception is that not all functional RNAs are translated into proteins (see non-coding RNAs below).

### The genetic code

The genetic code is the set of rules used by living cells to translate the information encoded within genetic material into proteins. When DNA and RNA were first discovered, the relative simplicity of nucleic acids led many scientists to doubt that it carried the genetic information. DNA only has four different kinds of bases; the question was how it could code for 20 amino acids. If there were a 1:1 correlation between bases and amino acids DNA could only encode four amino acids. Pairs of bases would give 16 possible combinations which is still not enough. However if you consider a triplet code you have 64 possibilities, which is more than enough. This is the code that we are familiar with where each codon, a sequence of three nucleotides, specifies a particular amino acid. This triplet code still did not seem logical because now you have far more codons than you need. There are some other important questions about the genetic code too; are the spare codons used? Is the code overlapping? And is it continuous or are there spacers indicating the end of each codon?

Table 1 shows the genetic code as we now understand it. It is written as RNA with a U rather than a T because it is RNA that cells translate into amino acids. The code is said to be redundant or degenerate because a single amino acid is often coded for by more than one codon. In most cases it is the third nucleotide in the codon that differs; this is often referred to as the degenerate position.

**Table 1:** The genetic code

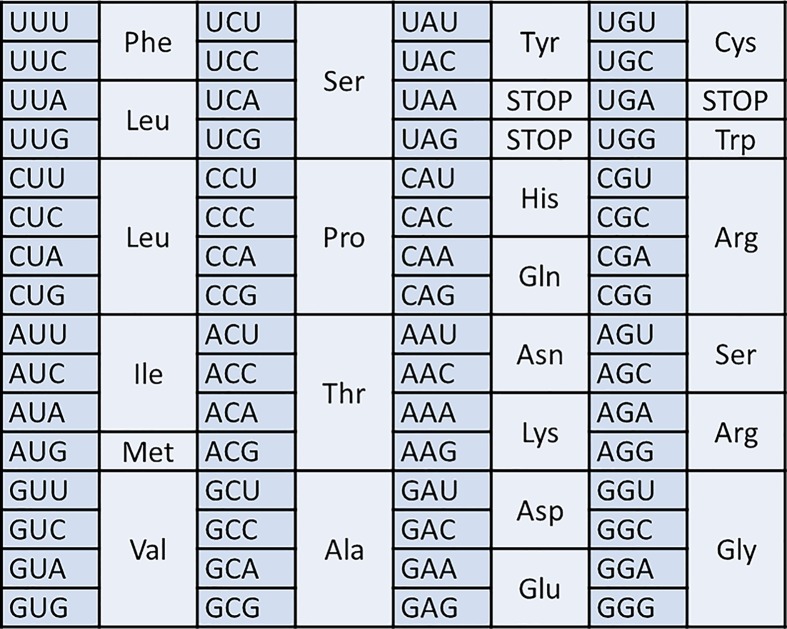

### Evidence for the triplet code

The experiments that allowed scientists to decipher the genetic code were carried out long before we were able to determine the sequence of DNA. While it was possible at that time to determine the proportions of each different amino acid in a protein, it was not yet possible to work out the order in which they occurred. Francis Crick and Sydney Brenner answered some key questions with an experiment using mutants of a virus that infects bacteria called bacteriophage. The normal or wild-type phage will infect *E. coli* and grow. Crick and Brenner investigated mutants that would not grow on some strains of *E. coli*.

**Figure 9:**
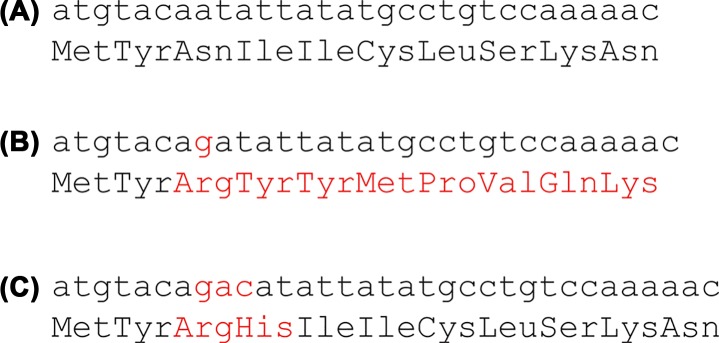
DNA sequence showing the amino acid translation underneath (**A**) Wild-type sequence, (**B**) a single base insertion (shown in red) causes a frameshift so all subsequent amino acids are different from the wild-type, (**C**) insertion of three base pairs (shown in red) causes two incorrect amino acids to be incorporated into the protein but there is no frameshift so the rest of the protein has the wild-type sequence.

Mutants which are insertions or deletions cause what are called frameshifts. Inserting a single adenine base into the DNA sequence not only changes the amino acid at the position of the insertion but all subsequent amino acids translated from that sequence (Compare Figures 9A and B); the reading frame has been shifted by one base and it results in a protein that is non-functional. However if you insert three nucleotides you often get a wild-type or near wild-type phenotype. This is because you have inserted a whole triplet codon, you will get one or two amino acids that were not in the original sequence but the reading frame is not shifted (Figure 9C) and the rest of the sequence is normal.

Crick and Brenner were looking for what they called suppressor mutations that would rescue the mutant and allow it to grow normally. They showed that their suppressor mutants did not simply reverse the original mutation; they often added or subtracted one or more bases. They worked out that if you insert or delete one, two or four nucleotides then you see a mutant phenotype. However, if you insert or delete three nucleotides, this has little or no effect. This was strong supporting evidence for a triplet code. This is also evidence for a redundant code where the same amino acid can be coded in more than one way. If the code were non-redundant there would be 20 codons that code for amino acids and 44 that are ‘nonsense’ codons. In this case inserting three nucleotides would be most likely to introduce a nonsense codon and not restore the wild-type. Crick and Brenner proposed correctly that the genetic code is read from a fixed starting point and the bases are read in groups of three.

### Cracking the code

At about the same time two American scientists Marshall Nirenberg and Heinrich Matthaei had developed a cell-free system which could synthesise proteins in a test tube when provided with an RNA molecule. They showed that when provided with an artificial RNA chain composed only of uracil (polyuracil) the system made a polypeptide composed entirely of phenyalanine residues. They now had a tool that they could use to crack the genetic code. RNA composed of cytosine (C) residues directed the synthesis of polyproline and RNA composed of adenosine (A) made polylysine. Experiments with combinations of nucleotides demonstrated that, for example, if you make RNA from A and C you produce proteins containing only six amino acids: asparagine, glutamine, histidine, lysine, proline and threonine. There are eight possible triplet codons that can be made from A and C, two of these we know encode proline and cysteine. The remaining four amino acids must be encoded by other combinations of A and C. This of course provides additional evidence for the redundancy of the genetic code.

These experiments using RNA molecules composed of random combinations of two or three bases were not enough to fully crack the genetic code. The use of chemically synthesised RNA molecules of known repeating sequence added some more important information. For example a synthetic RNA of alternating A and G residues (AGAGAGAGAG…) can be read as two alternating codons CAC and ACA. It encodes a protein of alternating histidine and threonine residues.

In the last section, we will discuss how tRNAs and ribosomes decode the genetic code and synthesise proteins. The final detail of the genetic code was determined by a technique using ribosome-bound tRNAs. Pieces of RNA as short as a single codon will bind to ribosomes and if amino acids attached to tRNA are added they will associate with the complementary RNA. If you then filter the solution you trap only the tRNAs that are bound to the ribosome, these are the ones specified by the codon in your RNA.

### Start and stop codons

Of the 64 possible codons, 61 encode amino acids. The three remaining codons: UAA, UAG and UGA do not code for an amino acid, they are sometimes called nonsense codons. They are stop codons; when the ribosome encounters these protein synthesis stops. The AUG codon encodes the amino acid methionine but it is also the most common start codon. As you will see in the last section, the first residue in eukaryotic proteins is always a methionine and in prokaryotes it is a modified amino acid N-Formylmethionine.

### Expanding the genetic code

Nature uses a small set of amino acids to make proteins, however if we were able to engineer cells that could use a wider range of building blocks with different physical and chemical properties it would be possible to make novel materials some of which could have useful therapeutic properties; this is one of the aims of synthetic biology. To do this successfully we need to reprogramme the genetic code and to engineer the translation machinery (see later section) to use these new combinations. Some progress has been made, for example in using both the UAG stop codon and the AAG codon for arginine to code for amino acids not normally found in proteins.

### Current concept of the gene

Once the genetic code was cracked it was clear that a gene is a sequence of bases on a DNA molecule that codes for a sequence of amino acids in a polypeptide chain or for an RNA molecule with a specific function. The availability of DNA sequences (see ‘Recombinant DNA Technology and DNA Sequencing’ in this issue of *Essays in Biochemistry*) of individual genes made it possible to look for patterns characteristic of genes. A gene that codes for a protein has a start codon followed by a series of codons that encode the amino acid sequence and then a stop codon; this is called an open reading frame.

Whole genome sequencing has provided biological data on an unprecedented scale. The need to analyse sequence data has led to the development of the field of bioinformatics; the analysis of these data to answer biological questions. One key concept used in bioinformatics is that of homology. Two organisms that have a common ancestor are said to be homologous and the same can be said of a structure or of a gene. For example limbs with five digits (the pentadactyl limb) are found not only in humans and other mammals but also in birds, reptiles and amphibians. The limbs are homologous, and this is evidence of a common evolutionary ancestor of all of these groups of animals. The same is true of genes. All vertebrates have red blood cells that contain haemoglobin, adult human haemoglobin is made from two α and two β globin molecules. The DNA sequence of the genes that encode globin molecules in vertebrates are all similar to each other and you can estimate how long ago two animals shared a common ancestor by looking at how similar their globin genes are. This principle can also be used to find genes in a new piece of DNA sequence; if there is a section of sequence that is similar to a known gene then it is likely to encode a homologous gene.

**Figure 10:**
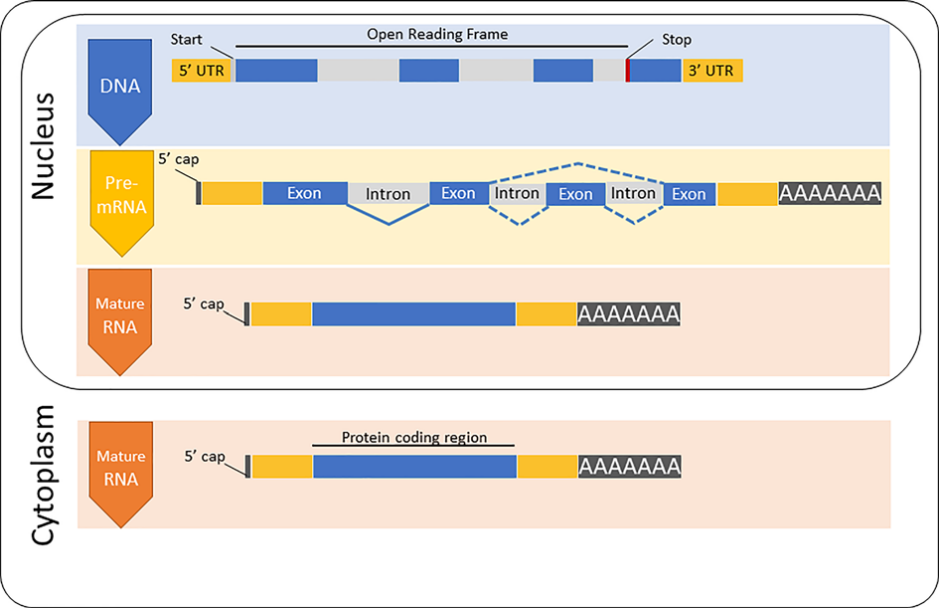
The structure of a protein-coding eukaryotic gene The DNA includes an untranslated region at both the 5′ and 3′ ends as well as introns and exons. The codon where translation starts (green) and the stop codon (red) are shown. The DNA is transcribed into mRNA and is processed by addition of the 5′ cap, splicing out the introns and addition of the poly A tail. This mature mRNA is exported from the nucleus into the cytoplasm.

A gene is more than just the sequence that encodes the protein; it also includes sequences involved in regulation of gene expression such as promoter sequences that define where transcription starts and are the sites where proteins involved in transcription bind to the DNA. In bacteria, almost all genes are a single uninterrupted sequence of DNA. In eukaryotes the situation is more complicated because the coding region is usually interrupted by introns. The primary transcript is referred to as precursor or pre-mRNA, this contains both exons and introns. The introns are removed when the pre-mRNA is processed before it leaves the nucleus (Figure 10) leaving the exons which are spliced together to make the mature mRNA. Eukaryotic mRNAs have a 5′ cap which is a methylated guanosine nucleotide added to the 5′ end of the mRNA by an unusual 5′ to 5′ linkage; this is important in initiating translation. At the 3′ end is the poly A tail, this is a chain of between 100 and 250 adenine residues added to the mRNA to increase its stability. Analysis of the human genome sequence suggests that there are approximately 20000–25000 protein-coding genes, however there are far more different proteins. This is because many genes are capable of encoding several variants of a protein. Alternative splicing allows for different combinations of exons to be included in the mature mRNA and genes can also have several alternative promoters and alternative poly A sites. It is thought that 95% of human genes are alternatively spliced.

### Non-coding RNAs

Only approximately 1.2% of the human genome codes for protein. However, if you compare the genomes of the human, the mouse and the dog you can see that much more of the genome is under what is called negative selection since the species diverged. Negative selection means that mutations which are disadvantageous are selected against. This suggests that more than just the protein coding regions affect the fitness of the organism carrying the DNA. Some of these are DNA sequences that are important in controlling gene expression (next section). However systematic screens are revealing large numbers of RNA transcripts that are processed but do not encode proteins. The most well-known are transfer RNAs and ribosomal RNAs both of which as we will see in a later section are fundamental to protein synthesis. However we are beginning to understand that there are other non-coding RNAs that carry out important cellular processes.

Two types of non-coding RNA, small inhibitory RNAs (siRNAs) and microRNAs (miRNAs) have a role in reducing gene expression after the mRNA has been transcribed from the DNA. They work by targeting a protein complex called RISC to specific mRNAs which it then degrades. Expression of the gene is specifically knocked out or reduced and the phenotypic effect of this can then be observed. Another group of non-coding RNAs play an important role in increasing the stability and correct folding of ribosomal RNAs. This process takes place in a compartment within the nucleus called the nucleolus; the RNAs are called small nucleolar RNAs (snoRNAs). These are mostly generated from intron RNA after it has been spliced out of the precursor mRNA and they function in association with proteins.

### Modern concept of a gene

The modern concept of the gene has to take into account all of the complexity of mRNA processing including alternative splicing, regulatory sequences and polyadenylation sites as well as the plethora of non-coding RNAs. A definition of a gene that takes these factors into account would be that a gene codes for one or more transcripts that can function as an RNA or can be translated into one or more proteins.

## Transcription

We have seen that a gene can encode either an RNA product or a protein sequence. The production of both requires the gene to be transcribed into RNA, either because the RNA is the final product or because the RNA will need to act as template for protein synthesis. RNA synthesis is very similar in prokaryotes and eukaryotes, being catalysed by the enzyme RNA Polymerase. However, of the processes discussed in this article it is arguably the one that differs most between prokaryotes and eukaryotes. One difference is that in eukaryotes the whole process needs to occur in a chromatin context, so access to the DNA template is limited. Regulation of gene expression is a major facilitator of cell differentiation, homoeostasis and speciation. Different cell types turn on transcription of different genes giving rise to their differentiated phenotypes. If we look at mammals as an example of speciation, they all have roughly the same gene content; it is how transcription is regulated that has changed as mammals have evolved. For example, if you compare humans and mice, the important changes to the human and mouse genome sequence that have occurred since they diverged from a common ancestor, are predominantly in the sequences that control transcription rather than in protein coding sequences.

### RNA polymerase

DNA-dependent RNA polymerases are responsible for transcription of DNA into RNA. Like DNA Polymerase, RNA polymerase requires a DNA template and nucleoside triphosphate precursors. RNA polymerase does not require a primer. During RNA synthesis, the base within the incoming nucleoside triphosphate pairs with the base on the DNA template, a phosphodiester bond is formed, and pyrophosphate is released. RNA polymerase synthesises RNA in the 5′ to 3′ direction, because it can only add nucleotides on to the 3′ end of the chain. During transcription only one DNA strand is transcribed into RNA.

### Gene transcription

**Figure 11:**
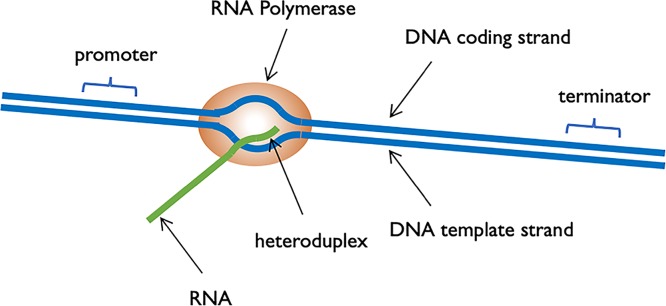
Schematic diagram of transcription

When a gene is transcribed, RNA polymerase will bind upstream from the start of the gene, it will unwind almost two turns of the DNA helix to form a transcription bubble, it will add nucleotides on to the growing RNA chain, the last 12 nucleotides to be added to the RNA chain will base pair with the DNA template, forming a DNA–RNA heteroduplex (Figure 11).

As each nucleotide is added to the growing chain, the transcription bubble and the heteroduplex moves with respect to the DNA template. So, as RNA polymerase synthesises RNA, there is unwinding of the DNA template in front of the site of synthesis and rewinding of DNA once RNA polymerase has passed through. Once RNA polymerase has transcribed the gene, transcription will terminate. For some genes, transcription termination is signalled by a particular sequence within the DNA, a terminator sequence, which RNA polymerase recognises. In some cases, RNA polymerase requires the help of other protein factors to recognise the terminator sequence. Finally, many eukaryotic genes do not contain a specific terminator sequence; instead, termination of transcription is linked to other events, for example cleavage of the RNA prior to addition of the polyA tail. Termination of transcription, leads to the dissociation of RNA polymerase from the DNA template and release of the RNA product. In prokaryotes, mRNA does not need processing before it can be translated, in fact, as will be discussed below, mRNA is translated as it is being made. However, the initial transcript in eukaryotes does need to be processed to produce a functional mRNA that can be exported to the cytoplasm for translation.

### Control of transcription in prokaryotes

**Figure 12:**
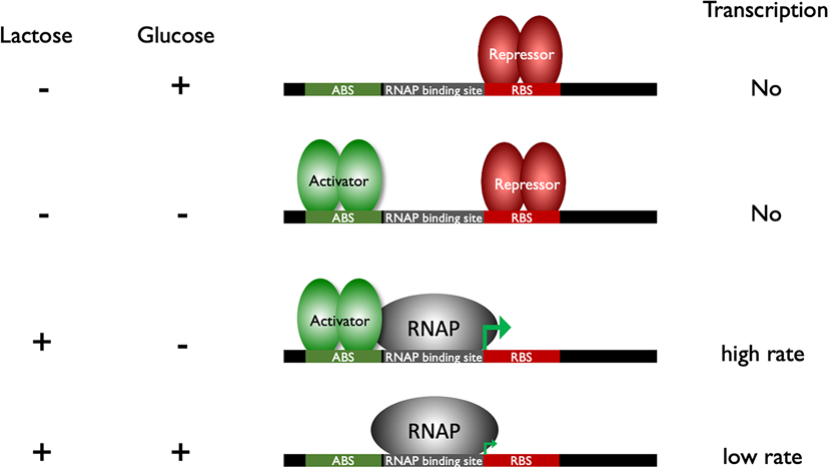
Control of transcription in prokaryotes The different binding sites for transcription factors are shown on the DNA; ABS, activator binding site, RBS, repressor binding site. The left-hand panel indicates the presence of lactose and/or glucose in the environment, the right-hand panel indicates transcription levels.

At many genes in prokaryotes, RNA polymerase can bind to the gene and initiate transcription without other protein factors. However, for most prokaryotic genes, the binding of RNA polymerase to the gene is controlled by transcription factors to ensure the correct genes are transcribed at the correct level within the cell. Upstream from the transcription start there will be a ‘promoter’ which contains specific DNA sequences that are recognised by RNA polymerase and transcription factors. Each gene will have a different promoter sequence and can be controlled by different transcription factors. A good example of this type of promoter is the promoter that controls the *lac* operon in *E. coli* (Figure 12). Transcription factors that up-regulate transcription are called activators and those that down-regulate transcription are called repressors. In this example RNA polymerase on its own can bind the promoter and drive low levels of transcription. If the repressor binds it will stop all transcription and would override RNA polymerase and the activator. In the absence of the repressor, if the activator is present then it can drive high levels of transcription.

The *lac* operon codes for genes required to use lactose and needs to be controlled in response to glucose and lactose concentrations. A repressor protein is responsible for responding to lactose concentration and an activator is responsible for responding to glucose (Figure 12).

In the absence of lactose, the *lac* operon is kept in an off state by the repressor protein binding to the promoter and stopping transcription. If lactose is present in the cell, it will bind to the repressor and this stops the repressor binding the promoter, RNA polymerase can bind and drive low levels of transcription. If the cell is starved of glucose, the activator is turned on and this binds the promoter and helps RNA polymerase to initiate transcription, resulting in high rates of transcription.

In the examples above, RNA polymerase on its own drives low levels of transcription. This might not be the case for all promoters, at some promoters RNA polymerase may not be able to bind and drive transcription without an activator protein. At other promoters RNA polymerase on its own will be able to drive high levels of transcription and a repressor protein would be needed to turn off transcription.

### Control of transcription in eukaryotes

**Figure 13:**
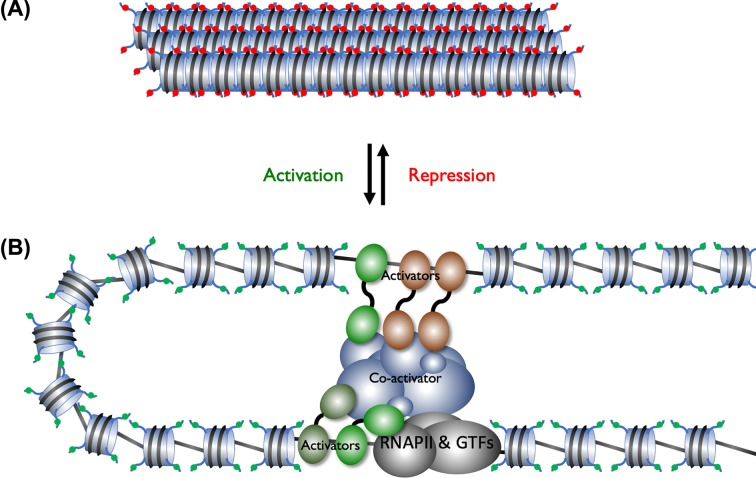
Regulation of transcription in eukaryotes (**A**) When a gene is in a silent state the surrounding DNA will be in condensed chromatin and the histones will epigenetic modifications which facilitate gene repression (red spheres). (**B**) A gene that is being transcribed will have activators bound to enhancer sequences, the activators recruit co-activators that acetylate the histone and add other epigenetic modifications that facilitate gene transcription (green spheres). The activator and co-activators will recruit RNA Polymerase and the GTFs to the core promoter.

Control of transcription in eukaryotes has to occur on a chromosome which is condensed into chromatin (Figure 13). In addition, transcription requires the assembly of a large multiprotein complex at the gene. This complex will contain RNA polymerase and several other general transcription factors (GTFs). The core promoter is a region that overlaps the transcription start and is the binding site for RNA Polymerase and the GTFs. In addition, there will be further control sequences, enhancers, that can be just upstream or several 1000 base pairs away from the core promoter. In the absence of activator proteins the chromatin structure will stop RNA polymerase and the GTFs binding to the core promoter. Here histone proteins act as generic repressors of transcription. In order for a transcription to be turned on activators will bind the enhancers and recruit co-activators which open up the chromatin structure and ensure the core promoter is not blocked by histone proteins. The activators and co-activators will then assemble RNA polymerase and the GTF at the core promoter and drive transcription initiation. Transcription factors will also ensure the chromatin structure across the whole gene is in a conformation that is suitable for transcription.

Repressors are not normally required to block assembly of transcription complex at the core promoter, however, they are important in the regulatory patterns needed in complex multicellular organisms. Eukaryotes have repressor proteins which can block the action of a specific activator and ensure the activator is only active when required. Repressors can work in a number of ways including binding to DNA and blocking the binding of the activator to the DNA, stopping the activator interacting with other proteins required for transcription or by binding to the activator and keeping the activator in the cytoplasm.

### Epigenetics

As discussed above transcription initiation in eukaryotes requires the opening up of the chromatin structure. This is facilitated by co-activator proteins that can move the relative position of the nucleosomes (Figure 4) with respect to the DNA and hence make certain regions of the DNA more accessible. They can also add chemical tags to both the histone proteins and DNA (Figure 13). These epigenetic modifications can affect whether a gene or genomic region is available for transcription or is transcriptionally silenced. Histones are acylated by enzymes which transfer an acetyl functional group to from acetyl-coenzyme A to lysine residues in the histone protein. This is linked to activation of transcription because it reduces the positive charge on histones and therefore reduces their affinity for the negatively charged DNA. Acetylation can also act as a tag that is recognised by other proteins that drive gene transcription. This modification of the DNA is described as epigenetic because it affects gene expression rather than the genetic code itself. Conversely some repressor proteins will recruit co-repressors that deacetylate histones, increasing their affinity for DNA causing the chromatin to be highly condensed and leading to transcriptional silencing. Methylation of lysine residues is another epigenetic tag, a single lysine residue can have 1, 2 or 3 methyl groups added. Unlike acetylation, methylation of lysine residues does not change the positive charge. The consequences of histone methylation are more complex because depending on which lysine residue is methylated and the level of methylation, the tag may mark that region of the genome for transcription activation or repression.

DNA methylation is another important epigenetic modification which leads to transcriptional silencing of the genomic region that has been methylated. During differentiation in the developing embryo whole regions of the genome will be methylated and therefore transcriptionally silenced. The DNA methylation patterns are maintained during cell division and future generations of that cell.

### Analysing transcription on a global scale

For many years individual scientists would study the transcriptional regulation of their ‘favourite’ gene and so we gained an understanding of how individual genes were regulated in response to different development or environmental signals, for example the control of the *lac* operon in response to lactose and glucose. In the last 15 years, many techniques have been developed to allow us to study transcriptional control of genes within a cell. Using techniques such as ‘RNA-Seq’, we can isolate the total RNA from a cell and use high-throughput sequencing to catalogue the level of transcription of all genes. In the case of eukaryotes this will also show how they have been spliced. It is also possible to analyse the binding of transcription factors and study epigenetic changes within histone proteins across the genome using techniques such as ChIP-Seq. So, combining techniques such as RNA-Seq and ChIP-Seq we can determine when and where a protein factor is bound to DNA and study epigenetic changes in a particular cell type and the consequences in terms of gene transcription. In combination these techniques give a detailed picture of the factors that affect transcription; this has been used, for example to look at differences between cancer cells and normal cells from the same patient.

### Transcription and disease

Transcription factors and promoters play major roles in health and disease, below are just a few examples to give an idea of their role in health and disease.

- The transcription factor p53 is a tumour suppressor protein, it guards against cancer and some human cancers have mutations that knock out p53 function.
- The drug Tamoxifen used in the treatment of breast cancer binds the oestrogen receptor inhibiting its function. The oestrogen receptor is a transcription factor that turns on the transcription of genes in response to oestrogen.
- Rett Syndrome is a neurodevelopmental disorder that affects approximately 1 in 15000 female births. It is due to mutations in a transcription factor that would normally repress transcription of specific genes, the mutations lead to inappropriate transcription of these genes.
- Cocaine use results in changes in expression of many genes, this can include epigenetic changes within genes involved in cognition and brain function. These epigenetic changes can be inherited and there is evidence that cocaine use by a father can result in epigenetic changes that result in male, but not female, offspring being cocaine resistant.

## Translation of RNA into proteins

The key player in protein synthesis is the ribosome, a complex structure composed of RNA and proteins. The ribosome provides a framework that ensures that the mRNA and tRNA are correctly positioned enabling the deciphering of the genetic code. There are many other proteins that are important in protein synthesis; some of these are part of the ribosome and some are again correctly positioned by the framework of the ribosome. As we will see, the small subunit ribosomal RNA is a ribozyme; an RNA molecule with catalytic properties similar to those of enzymes. Ribosomal RNA can form a peptide bond between two amino acids.

### Transfer RNA

**Figure 14:**
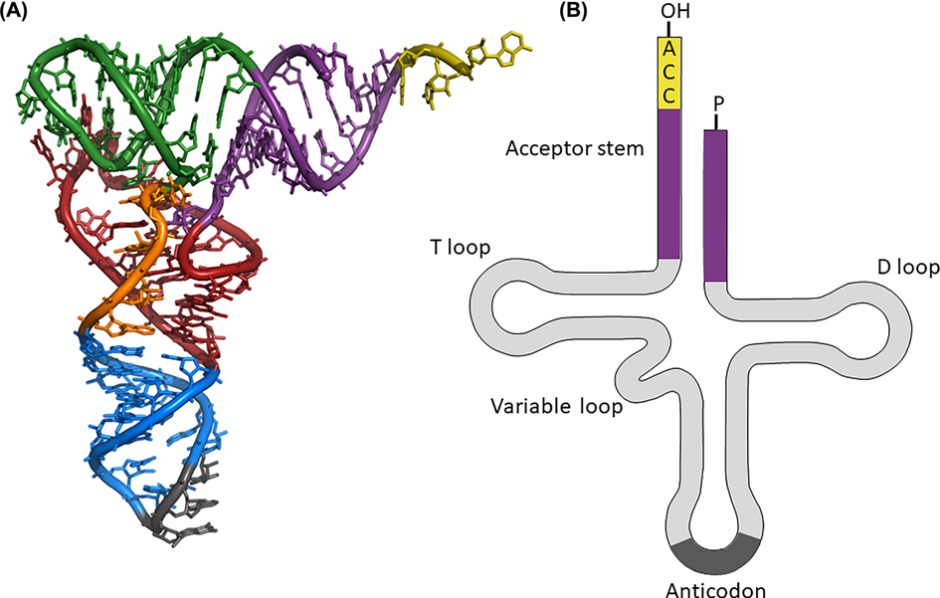
Transfer RNA (**A**) Tertiary structure of the phenylalanine tRNA from yeast showing the anticodon (grey), the acceptor stem (violet) with the nucleotides CAA at the 3′ OH end (yellow). Image modified from ‘TRNA-Phe yeast’ Yikrazuul (licensed under CC BY-SA 3.0). (**B**) Clover leaf representation of the secondary structure of tRNA.

The other nucleic acid that you need for protein synthesis is the tRNA. The tRNA molecule is single stranded and folds up into a characteristic structure by base pairing (Figure 14). These act as adaptor molecules, each has an anticodon for a specific mRNA codon and each carries the amino acid specified by that codon. The anticodon has a complementary sequence to the codon on the mRNA.

The enzymes which attach amino acids to tRNAs are called aminoacyl tRNA synthetases; they recognise a specific amino acid and the corresponding tRNA. The reaction also requires ATP, it is carried out in two steps:


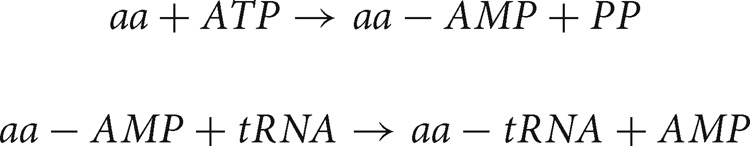


In the first step the enzyme hydrolyses ATP releasing pyrophosphate (PP) and in the second it attaches the amino acid to the 3′ hydroxyl of the tRNA. Aminoacyl tRNA synthetase enzymes are highly specific, they recognise specific amino acids and will only attach them to the correct tRNA. This ensures correct coupling of amino acids and tRNA molecules which is just as important in ensuring the fidelity of protein synthesis as the matching of the anticodon to the codon by the ribosome. In addition this step is said to activate the aminoacyl tRNA as it not only produces the correct substrate for the ribosome but also provides much of the energy required for peptide bond formation during protein synthesis.

### Structure of the ribosome

**Figure 15:**
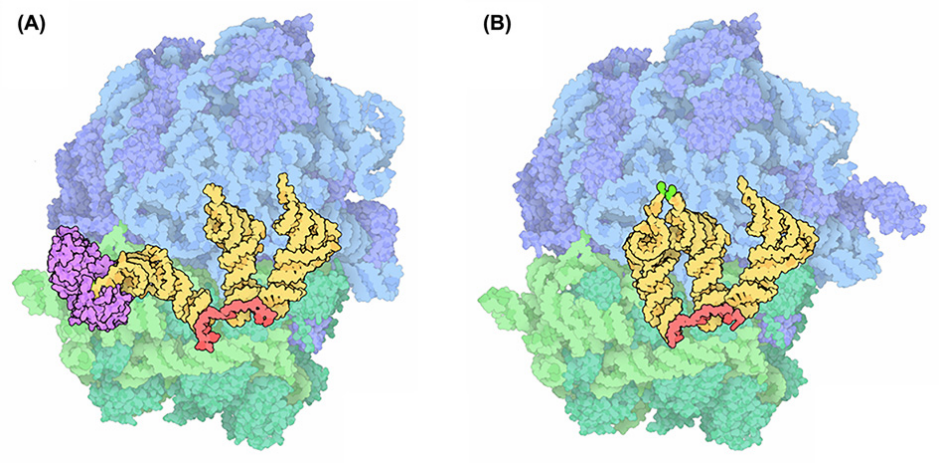
The structure of the ribosome of *Thermus thermophilus* showing the small subunit (green), large subunit (blue), mRNA (red) and three tRNAs in the acceptor, peptidyl and exit sites (yellow) In (**A**) the new tRNA is delivered to the ribosome by elongation factor EF-Tu (purple). In (**B**) the amino acid on the incoming tRNA is brought close to the amino acid on the tRNA in the peptidly site to facilitate peptide bond formation (bright green) (Adapted from Goodsell 2010, licensed under CC-BY-4.0 licence).

All living things contain ribosomes. The ribosomes in bacteria are slightly smaller than those found in eukaryotic cells (Table 2) but the overall structure and the way in which they work are essentially the same. The 2009 Nobel Prize for Chemistry was awarded to three scientists, Ada Yonath, Thomas Steitz and Venkatraman Ramakrishnan, who used X-ray crystallography to solve the three-dimensional structure of the bacterial ribosome. The ribosome is composed of two subunits, the small subunit which reads the messenger RNA and the large subunit which forms the bonds between amino acids, adding them to the growing polypeptide chain. There are three important binding sites for tRNAs in the ribosome which are at the interface between the two subunits and only formed when the two subunits come together. These sites are shown on the image in Figure 15, they are referred to as the acceptor or aminoacyl (A) site, the peptidyl (P) site where the peptide bond between amino acids is formed and the exit (E) site from which spent tRNAs leave the ribosome.

**Table 2:** Properties of ribosomes

	Bacterial ribosome	Eukaryotic ribosome
Size	70S*, 2.3–2.6 MDa	80S, 3.3–4.5 MDa
Small subunit	30S	40S
	Approximately 20 proteins	32 proteins
	16S rRNA, ∼1600 nucleotides	18S rRNA
Large subunit	70S	60S
	Approximately 33 proteins	79–80 proteins
	23S rRNA, ∼2900 nucleotides	25S rRNA
	5S rRNA, ∼120 nucleotides	5.8S & 5S

* S stands for the Svedberg unit for sedimentation velocity.

In addition to the ribosome, the mRNA and tRNA, there are a number of small proteins that are not part of the structure of the ribosome, but are required for protein synthesis: initiation factors, elongation factors and termination factors. The importance of these factors is illustrated by the inherited condition Vanishing White Matter Disease (VWM). This serious neurodegenerative disease which results in lesions in the white matter in the brain is due to mutations in one of the initiation factors.

### Protein synthesis

During protein synthesis the ribosome brings together the amino acid charged tRNA and the mRNA, the codon and anticodon are matched and the amino acids are joined together in the correct sequence. There are three phases to this process: initiation where the ribosome assembles on the mRNA, elongation where the triplet code is read and amino acids are added to the growing peptide chain and termination where protein synthesis stops.

#### Initiation

**Figure 16:**
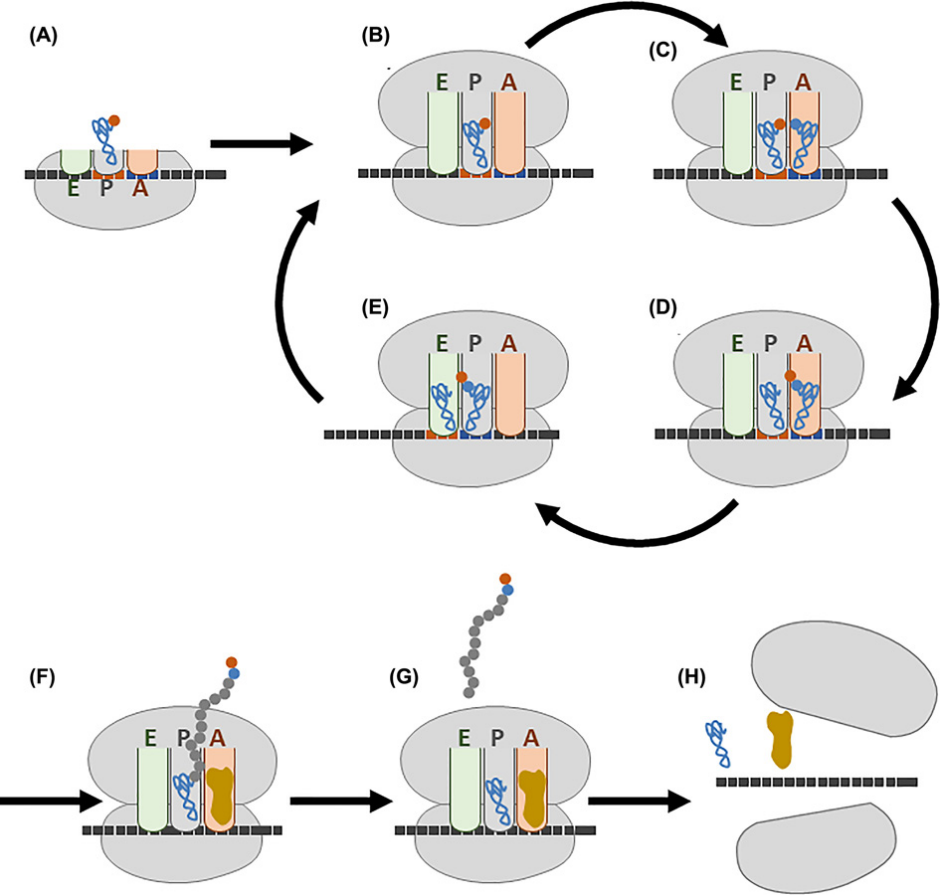
Protein synthesis (**A**) During initiation, the mRNA recruits a tRNA charged with a methionine and the small ribosomal subunit, (**B**) the large subunit then docks to give the translation complex, (**C**) a tRNA with an amino acid attached enters the A site, (**D**) the peptide bond is formed between the amino acid in the P site and the one in the A site. The effect is that the growing peptide chain is transferred to the incoming aminoacyl tRNA in the A site leaving an empty tRNA in the P site. (**E**) Finally, everything moves along the mRNA by one codon in a process called translocation so the peptidyl tRNA with the growing peptide chain attached moves to the P site and the spent tRNA to the E site from where it leaves the ribosome. (**F**) When a stop codon is in the A site, a termination or release factor enters the A site, (**G**) the peptide is released from the ribosome and (**H**) the two subunits of the ribosome disassociate and are recycled.

A complex of proteins called the cap-binding complex bind to the 5′ cap of the mRNA (Figure 10) in the nucleus. The mRNA is then exported to the cytoplasm where it recruits initiation factors, tRNA charged with a methionine and the small (40S) ribosomal subunit. Initiation factors also bind and the small subunit scans along the 5′ untranslated region of the mRNA until it encounters the first AUG start codon (Figure 16A). This is recognised by the anticodon codon of the initiator tRNA, the large subunit then docks to give the translation complex. The 80S ribosome with the tRNA charged with methionine at the P site is now ready to accept the next tRNA (Figure 16B).

#### Elongation

**Figure 17:**
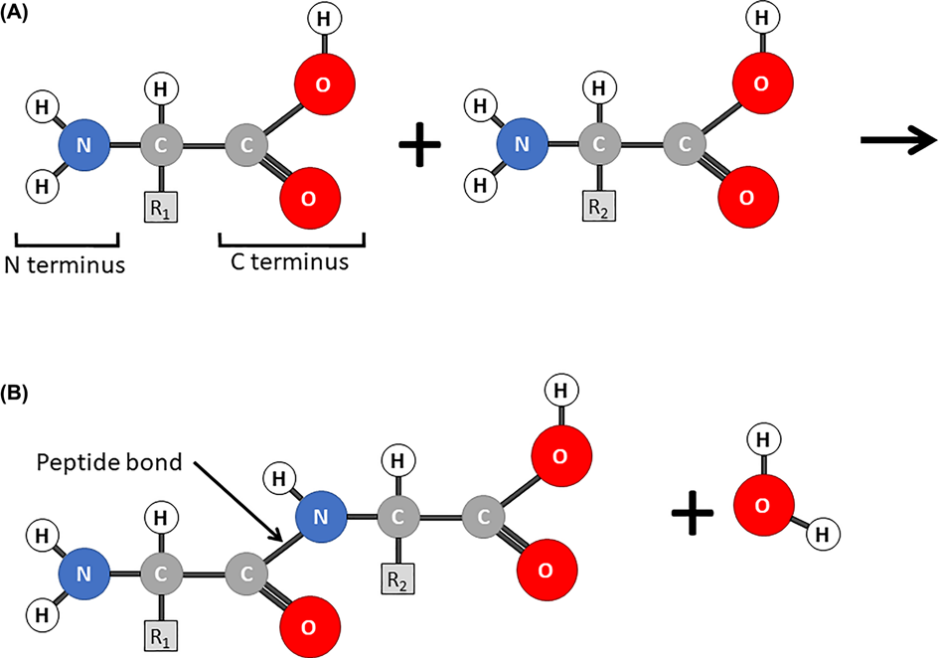
Amino acids and peptide bonds (**A**) Amino acids consist of a carbon atom with an amine group (the N terminus), a carboxylic acid group (the C terminus) and a variable R group. The simplest R group is a methyl group giving the amino acid alanine. (**B**) When two amino acids are joined together a peptide bond is formed between the N terminus of one amino acid and the C terminus of another. This is a condensation reaction releasing one molecule of water.

With initiation complete, the mRNA is in the correct reading frame with the A site empty and the next codon exposed. In the elongation phase an aminoacyl tRNA, one charged with an amino acid, is brought to the ribosome in a complex with an elongation factor and enters the A site. If the anticodon it carries is complementary to the exposed codon it is correctly positioned in the acceptor site and GTP is hydrolysed on the elongation factor (Figure 16C). A peptide bond (Figure 17) is then formed between the C terminus of the amino acid in the P site and the N terminus of the amino acid in the A site, this reaction is catalysed in the peptidyl transfer centre of the large subunit of the ribosome. The effect is that the growing peptide chain is transferred to the incoming aminoacyl tRNA in the A site leaving an empty or spent tRNA in the P site (Figure 16D). Finally, the peptidyl tRNA with the growing peptide chain attached moves to the P site. This step is called translocation and the energy being provided by hydrolysis of GTP by the elongation factor EF-G. The spent tRNA moves to the exit site from where it can leave the ribosome. The mRNA moves so that the next codon is exposed in the A site (Figure 16E) ready to accept a new aminoacyl-tRNA charged with another amino acid. During the elongation phase the ribosome cycles through this process, adding amino acids to the growing peptide chain until a stop codon is exposed in the A site. The new protein emerges from the ribosome through an exit tunnel in the large subunit.

#### Termination

The stop codon is not decoded by being recognised by an anticodon on a tRNA. Instead it is detected by proteins called termination or release factors. In eukaryotes there is a single release factor (RF1) that recognises all three stop codons enters the A site (Figure 16F). The ester bond linking the peptide chain to the tRNA in the P site is broken and the peptide is released from the ribosome (Figure 16G) The two subunits of the ribosome disassociate and are recycled (Figure 16H).

The structure and function of ribosomes are highly conserved with a large core of structurally conserved proteins and rRNAs found in both eukaryotic and prokaryotic ribosomes. However, there are some differences both in the rRNAs and in some of the additional proteins involved in translation (Table 2). The elongation phase is highly conserved but there are important differences in how protein synthesis is initiated. Bacterial mRNAs have a specific sequence called the ribosome binding site or Shine–Dalgarno sequence. In order to ensure that the mRNA is correctly positioned in the ribosome the Shine–Dalgarno sequence binds to a complementary sequence of the 16S rRNA in the small subunit. In bacteria the initiator tRNA is charged with a modified amino acid N-Formylmethionine.

Differences between the structure of bacterial and eukaryotic ribosomes can be exploited by antibiotics which are selective in that they affect protein synthesis in bacteria but not in mammalian cells. Macrolide antibiotics like erythromycin, block the exit tunnel in the large subunit of bacterial ribosomes and halt protein synthesis. The exit tunnel in eukaryotic ribosomes is slightly narrower which means that eukaryotic ribosomes are not affected. Streptomycin, an important antibiotic in the treatment of tuberculosis binds to the 16S of bacterial ribosomes. This distorts the structure of the decoding site and results in misreading of the mRNA.

### Polyribosome

**Figure 18:**
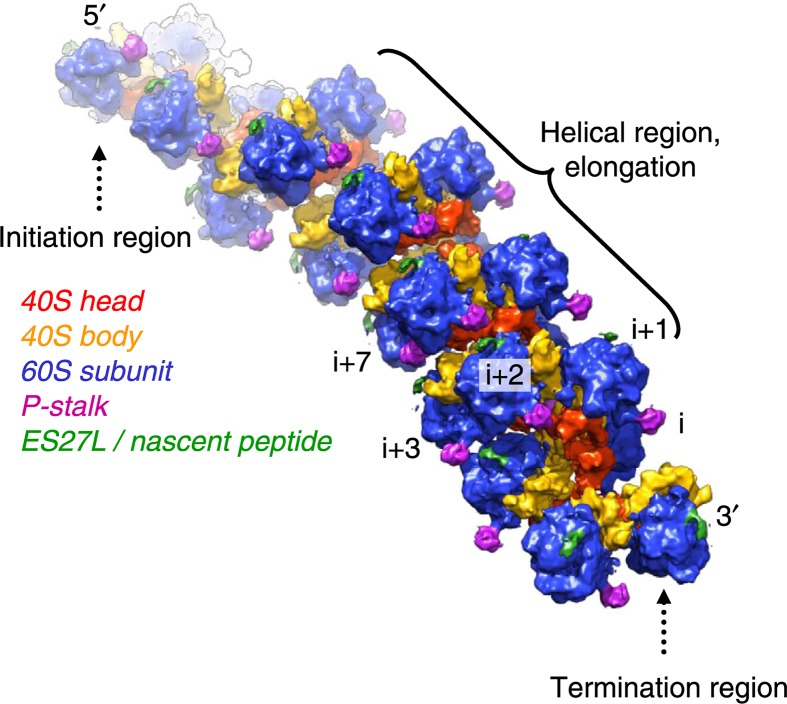
The polyribosome Cyro-electron micrograph reconstruction of eukaryotic polyribosome. Reprinted from (Myasnikov 2014) by permission.

Protein synthesis can proceed very quickly, particularly in rapidly growing cells or those that are differentiating. In bacteria between 15 and 20, new peptide bonds can be formed per second. In eukaryotes it is slower, more like five peptide bonds per second. A small human protein like insulin would take only 10 seconds to make whereas the largest human protein titin, which is found in human muscle cells, takes about an hour and a half per molecule. One of the mechanisms that ensures that protein synthesis is carried out efficiently is the polyribosome. As soon as one ribosome has started translation another ribosome binds to initiate synthesis of another protein copy. This gives rise to polyribosomes or polysomes which can be seen by electron microscopy. Recent cryo-EM images show that ribosomes can be arranged very closely on the mRNA with the mRNA entry and exit channels aligned to allow the smooth passage of mRNA between them (Figure 18). Sometimes these polyribosomes can form circular structures so that, as soon as the ribosome has finished synthesis of one polypeptide it can rebind the same mRNA molecule and start synthesis of another copy of the protein.

### Closing remarks

The study of nucleic acids, from their first identification as the genetic material is littered with landmarks in molecular biosciences, many of them marked with Nobel Prizes. Since Watson and Crick proposed their structure of DNA our knowledge about DNA and how it works has expanded almost exponentially. The topics introduced in this article are important topics covered in all bioscience programmes; understanding them is key to all areas of biosciences from evolution and animal diversity to health and disease. Recent developments in the techniques that we can use to study DNA, often in living cells means that new and exciting developments in our understanding of the way nucleic acids work are occurring all the time. Given the scope of this article we have barely scratched the surface of the topic, however, the reader can find more detail from the articles in the bibliography below and even more detail from a few minutes searching on the internet.

## Abbreviations

DNA: deoxyribonucleic acid
GTF: general transcription factor
ori: origin of replication
RNA: ribonucleic acid
RISC: RNA-induced silencing complex
